# Transition Activity Recognition System Based on Standard Deviation Trend Analysis

**DOI:** 10.3390/s20113117

**Published:** 2020-05-31

**Authors:** Junhao Shi, Decheng Zuo, Zhan Zhang

**Affiliations:** Department of Computer Science and Technology, Harbin Institute of Technology, Harbin 150001, China; shijunhao@hit.edu.cn (J.S.); zz@ftcl.hit.edu.cn (Z.Z.)

**Keywords:** human activity recognition, transition activity, smartphone, SVM, trend analysis

## Abstract

With the development and popularity of micro-electromechanical systems (MEMS) and smartphones, sensor-based human activity recognition (HAR) has been widely applied. Although various kinds of HAR systems have achieved outstanding results, there are still issues to be solved in this field, such as transition activities, which means the transitional process between two different basic activities, discussed in this paper. In this paper, we design an algorithm based on standard deviation trend analysis (STD-TA) for recognizing transition activity. Compared with other methods, which directly take them as basic activities, our method achieves a better overall performance: the accuracy is over 80% on real data.

## 1. Introduction

Human activity recognition (HAR) is a significant subfield of pervasive computing and provides important context information for an ocean of applications such as medical care, education, and entertainment [1,2,3]. In recent decades, with the popularity of smart phones and other wearable devices, HAR applications based on built-in inertial sensors have been significantly developed. For example, WooSeok Hyun et al. [4] developed a wireless body sensor network which integrated different physiological sensors to sense physiological data from a human body with smartphones as computing platform. Chetty Girija et al. [5] realized an automatic and intelligent daily activity monitoring application for elderly people using smartphone inertial sensors. Espinilla Macarena et al. [6] designed a subwindow-based online recognition architecture, which achieved promising results in their experiments. Garcia-Ceja et al. [7] used sound and accelerometer data collected with a smartphone and a wristband while performing home task activities, and the whole system performed outstandingly using a multi-view stacking method. Zahin et al. [8] proposed a semi-supervised classifier, mainly using deep learning networks, in sensor-based smart health monitoring. In summary, most researchers pursue higher activity recognition accuracy.

**Figure 1:**
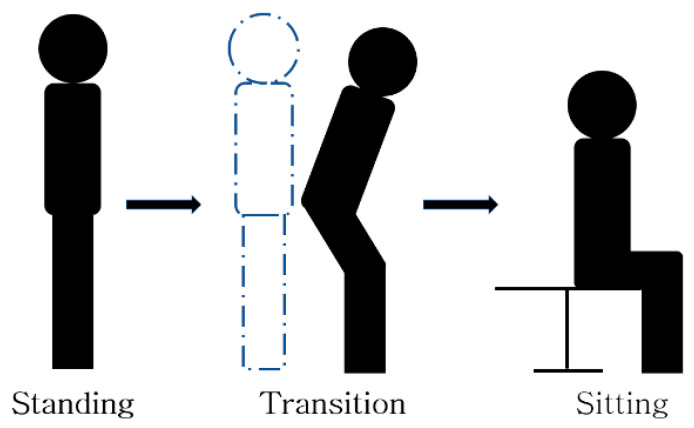
Example of transition activity.

Although most related works achieve excellent results in recognizing daily activities (e.g., walking, sitting, standing et al.) [9,10], there are still issues in HAR systems that affect its performance. One of them is transition activity, which is the transitional process between two different basic activities, as Figure 1 depicts.

Unlike basic daily activities, transition activity is usually transient and accompanied with intense changes. To the best of the authors’ knowledge, most related works choose to ignore the existence of these activities. Sometimes it may be an effective measure for specific practical needs. However, this will inevitably affect the performance of the HAR systems. HAR systems are faced with random noise in the real scenario, which obviously affects the performance of the overall system. Transition activity recognition helps to segment different basic activities to reduce the error rate. Moreover, some HAR systems focus on identifying the transitional activities, such as fall detection systems, require accurate recognition accuracy of these kind of activities. Therefore, transition activity recognition is necessary for HAR systems.

Several efforts attempt to solve this issue from their own perspectives. Reyes-Ortiz et al. [11] designed an architecture for recognizing transition activity. They used the probabilistic output of consecutive activity predictions as the main basis for identification and achieved promising results. Mohd et al. [12] took advantage of multivariate Gaussian distribution to judge whether the current activity is a transition activity. The above research mainly focuses on the transition between static activities, while there is relatively little discussion on transitions involving dynamic activities (e.g., standing↔walking).

In this paper, we build a HAR system based on smart phone built-in sensors. The overall work is depicted as follows:

1. We fuse two types of sensor data for accurately recognizing basic daily activities in our dataset.
2. We distinguish the transition activities from basic activities by analyzing the trend of standard deviation.
3. We develop an android application on a smartphone and conduct experiments in a real scenario.

The rest of this paper is organized as follows. In Section 2, we introduce related work, including HAR systems and approaches for recognizing transition activities. Section 3 gives the details of our proposed system architecture and algorithm. Section 4 covers the results of the experiment, and Section 5 is the conclusion and future work.

## 2. Related Works

### 2.1. Human Activity Recognition Systems

With the emergence of Micro-Electromechanical Systems (MEMS), researchers use a variety of professional sensing devices in the HAR systems. Pansiot et al. [13] developed the e-ar sensor, which can be worn on the ear to detect human body signs data for health care. Minnen et al. [14] put multiple sensors on a military suit to recognize tactical actions and provide battlefield information. Moreover, due to the popularity of smart phones, there are also studies developing HAR systems using smartphone built-in sensors. For example, Akhavian et al. [15] bound mobile phones to the upper arm of construction workers to recognize the workers’ ongoing activities. Wang et al. [16] developed a system to identify students’ behavior through a variety of built-in sensors in smartphones to evaluate their mental health and academic performance. Bisio et al. [17] used smart phones to realize telemedicine monitoring. Ronao et al. [18] proposed a two-stage continuous hidden Markov model (CHMM) approach for the task of activity recognition using accelerometer and gyroscope sensory data gathered from a smartphone. Lu et al. [19] designed an efficient and flexible framework for activity recognition based on smartphone sensors, and the proposed method was independent of device placement and orientation. Most similar HAR systems take advantage of smart devices for collecting sensing data and utilize machine learning algorithms (e.g., Support Vector Machine (SVM), Decision Tree (DT), K-Nearest Neighbor (KNN) [20,21,22]) for activity recognition. However, they choose to ignore the existence of transition activity instead of taking it as an important issue.

### 2.2. Transition Activity Recognition

For some studies, ignoring transition activity causes little influence on the result because they focus on long-term activity monitoring. However, there are also studies that address the transition issue. For example, Song et al. [23] used a 3D accelerometer for activity data collection, and they designed an event-based recognition architecture to deal with transition activity. In the method they utilized a hidden Markov model (HMM), which needed prior probability information, as classifier. Aminikhanghahi et al. [24] built a smart-home environment, placed various sensors on the human body and room doors to collect daily activity data and realized data segmentation and transition activity recognition through change point detection. This research mainly dealt with complex daily activities such as cooking. A similar scenario is also mentioned in Atallah et al. [25]. They used a method based on manifold embedding to map high-dimensional data into low-dimensional space. However, the recognition accuracy on standing-sitting was not high. Moreover, with the development of artificial intelligence, a quantity of works on transition activity regarded transition activity as a new kind of activity similar to the basic ones. Thien Huynh-The et al. [26] used SVM as a classifier to recognize transition activities. The approach was supported by excellent machine learning models. However, the transition activities can be easily classified as other activities in real scenarios, and vice versa.

In this paper, we refer to some ideas from the above works and identify transition activities by analyzing the standard deviation trend. As a simple recognition architecture, the system still achieves promising performance.

## 3. Method and Architecture

### 3.1. System Architecture

**Figure 2:**
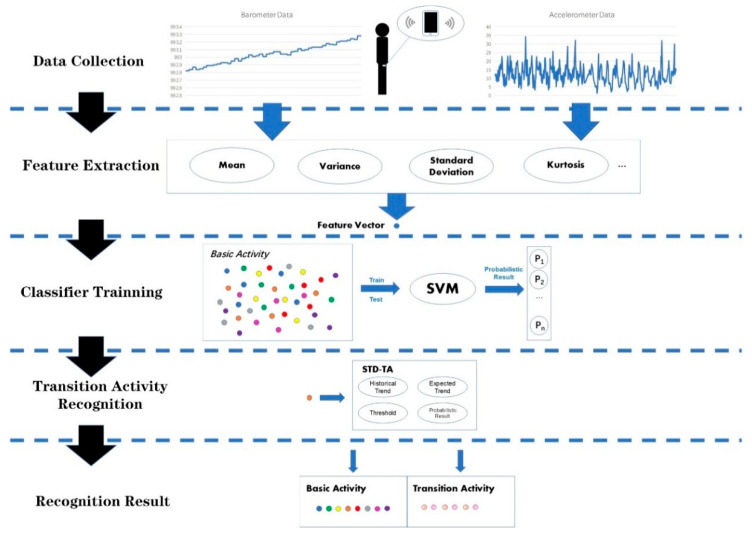
Overall activity recognition process.

In this part, we introduce the whole process of activity recognition, as shown in Figure 2.

First, based on the results of our previous work [27], we put a smart phone on the right leg of subjects to collect data in order to achieve the best classification results. The data includes accelerometer data and barometer data. We separate the set into two types: basic activity and transition activity. Then we segment data via a sliding window algorithm and divide all the segments into two parts, one for training and the other for testing. In the feature extraction stage, we extract the statistical features of data segments to reduce the dimension of data and facilitate the training of classifiers. Considering the excellent performance of SVM in pattern recognition [28,29], we train a SVM model as the core classifier in the classifier training stage. SVM outputs the recognition results in the form of probability, which is namely the probabilistic results. In the transition activity recognition stage, we take advantage of the standard deviation trend analysis (STD-TA) method to judge whether the current activity is a transition activity. The final results are achieved in the recognition result stage, and transition activity is distinguished from basic activity.

In the next parts, we give the details of each stage.

### 3.2. Data Collection

We collect eight simple daily activities in our work. Table 1 shows more information about our dataset.

Most activities can be recognized via processing the accelerometer data. According to our previous experience, we set the accelerometer data sampling frequency at 50 Hz, which is enough to achieve outstanding recognition accuracy. We gather the barometer data for recognizing different motion patterns with huge similarity, and 5 Hz is a proper sampling rate given that it changes little in a few milliseconds. For example, the characteristics of upstairs and downstairs in acceleration are highly similar, it is tough to distinguish these two activities unless taking the barometer data into consideration. This is also the general idea of studies on sensor fusion [30]. We apply an upsampling process to make every 10 accelerometer data samples correspond to the same 10 barometer samples to facilitate model training in the following stage.

We collect 3 min data for each activity of each subject (80 s for upstairs and downstairs). For dynamic activities, we ask the subject to perform the same activity for 3 min, and each group of data is equivalent to a periodic repetition of single activity. Based on the actual observation on the data curve, we finally determined the single duration of these activities. For transition activities, we ask each subject to repeat two basic activities within 3 min, and the sequence of activities we obtained might be like A, shown below:

A = {Walking, Transition, Standing, Transition, Walking, Transition, Standing}

We get about 10 transition activities per collection, which is enough for us to determine the duration of a single transition activity with observation. We make a statistic on the duration of these activities, as shown in Table 2. With the sliding window algorithm, we segment the original data into data segments, which represent single complete activities. The window size is determined by the duration of a single activity. Static activities such as standing and sitting are a long-term static state. We make a statistic on the duration of other activities, as shown in Table 2.

Compared with basic activities, the proportion of transition activities is relatively low in the real scenario. Therefore, the window size mainly depends on the basic activities, especially the duration of single dynamic activities. As shown in Table 2, the duration of dynamic activities is about 50 data points, and the 1 s time window can well cover all dynamic activities and part of the transition activities. For some tiny short transition activities, such as “Walking to Running”, it may fall into the same segment as some other dynamic activities. Given the transitional characteristic of this segment, we consider it as a transition activity. Based on the above information, we finally set the window size as 1 s (50 data sample points).

### 3.3. Feature Extraction & Classifier Training

Feature extraction can provide the classifier training with statistical characteristics of original data. We get four columns of data after data collection in every data segment, which are named as (Acc_x, Acc_y, Acc_z, Baro). At this stage, we extract statistic features, including mean, variance, standard deviation (STD), etc, of all data segments. More details are given in Table 3.

We define $Acc_{all}$ as

$$Acc_{all}=\sqrt{Acc\_x^{2}+Acc\_y^{2}+Acc\_z^{2}}$$

**Figure 3:**
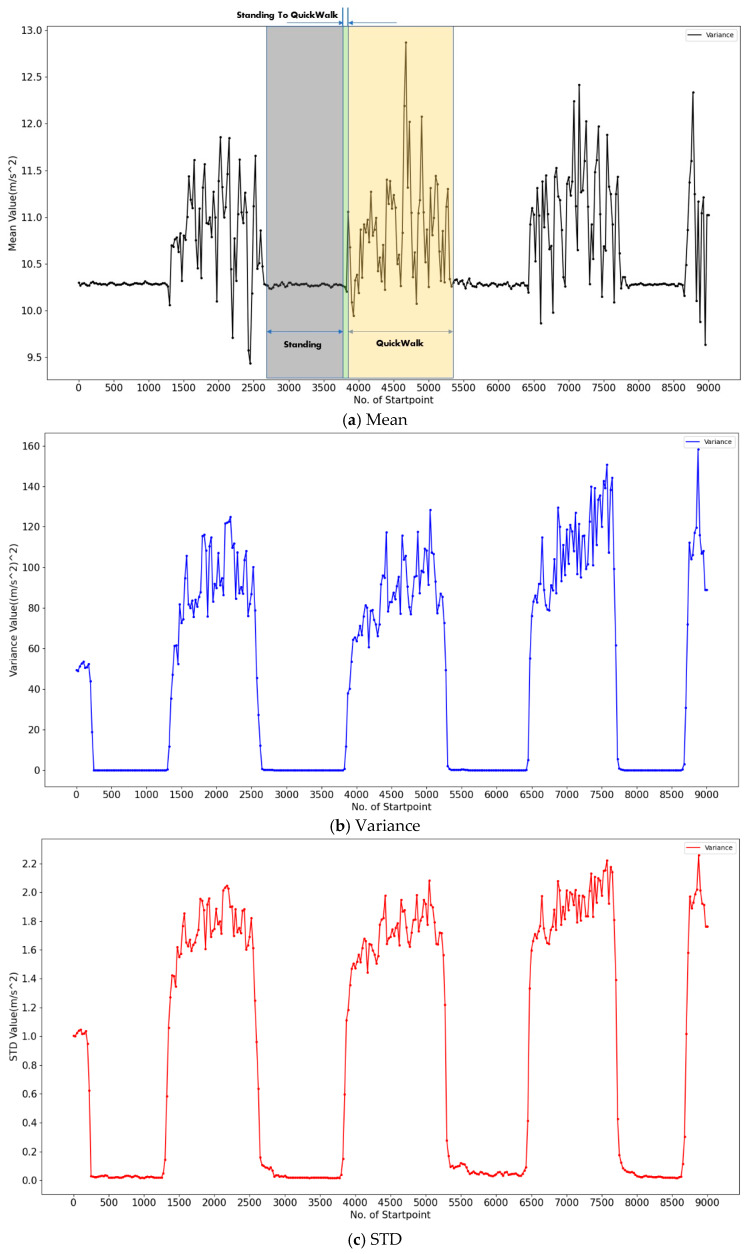
Mean, variance and STD of “Standing-QuickWalk”.

In this stage, we focus on the $Acc_{all}$ trend of transition activity data. Figure 3 shows the trend of three main features of “Standing-QuickWalk”.

Take Figure 3a as an example. The data in the gray area are acquired when the user is standing. There are little changes over this period, and the small area in the middle represents the user stopping “Standing” and beginning to “QuickWalk”, at which point the mean value increases significantly. Then, the data in the light-yellow area represent the user continuing to “QuickWalk”, and the mean value changes dramatically in this part. It is obvious that the mean trends of these two larger regions are hugely different, so they can be easily distinguished. We observe the value range of these three kinds of features in Figure 3. Mean is in range [9,13], variance in the range [0, 160] and STD in the range [0,2.2]. Compared with the first two features, the STD is smaller and more controllable, which is convenient for us to fine-tune. Therefore, we decide to use the STD trend analysis (STD-TA) as the transition activity recognition method.

On the other hand, we choose SVM as the classifier for basic activities. As a common model in pattern recognition, SVM performs well in a number of studies. It does not rely on a tremendous amount of data input, which is suitable for research with a very small dataset. In our work, the feature vectors formed in the feature extraction stage are divided into two parts, one for training and the other for testing. After training SVM, we deploy it into a real-time environment. Every real data segment produces a probabilistic vector **P** = {p_1_, p_2_,…,p_n_}, where p_i_ represents the probability that the data segment belongs to the ith activity. We name **P** as the probabilistic result, which is a vital basis to judge whether the current activity is a transition one.

### 3.4. Transition Activity Recognition and Recognition Result

**Figure 4:**
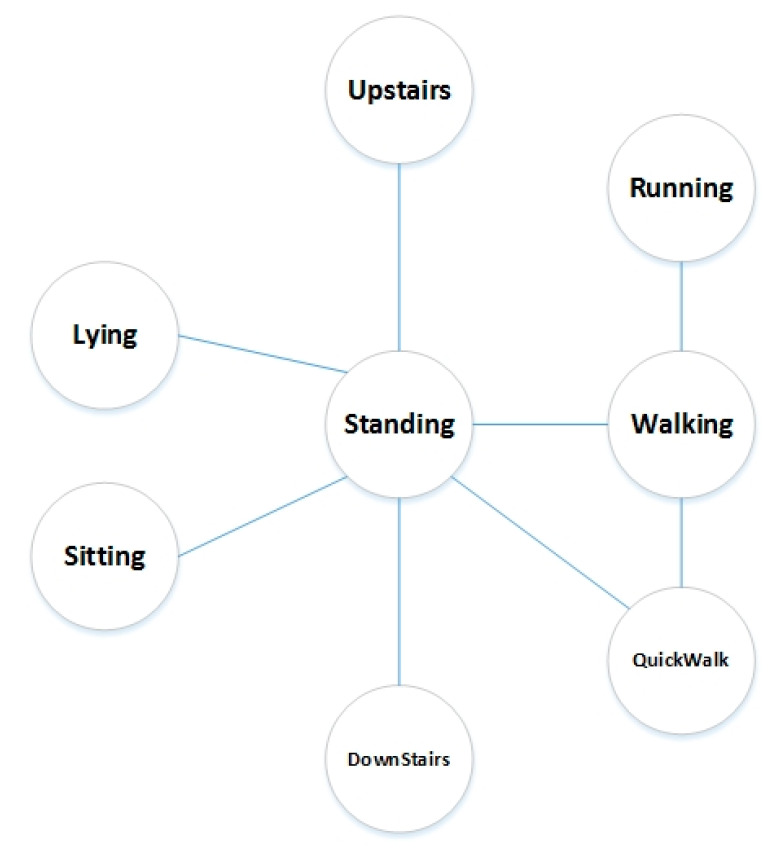
Activity transition diagram.

At this stage, we introduce the details of the STD-TA. First, we define the transition relationship of activities to determine where the transition activity can happen. We design a transition diagram, as Figure 4 shows.

Figure 4 mainly specifies the basic activities between which the transition activities will take place, so that some illogical transitions could be effectively avoided to promote the overall accuracy. In the figure, there is no transition between basic activities without connection, such as “Lying” and “Running”. The rules can effectively avoid some recognition errors. According to the diagram, there are 14 transition activities in total. Here, we clarify some definitions:

**Definition** **1.** **FVList** = {V_1_,V_2_,…,V_n_}

*where* V_i_ *is the feature vector extracted from the i*th* segment of a data sequence.*

**Definition** **2.** **P** = {SVM(V_1_), SVM(V_2_),…, SVM(V_n_)},

**PredAct** = {index(max(P_1_)),index(max (P_2_)),…, index(max(P_n_))}

*where* P_i_ = SVM(V_i_) ∈ **P** *denotes the probabilistic results of* V_i_ *after being classified by *SVM*,* PA_i_ = index(max(P_i_)) ∈ **PredAct** *is the category result of* V_i_, *which is the label with the maximum probability. We extract the *STD* value of* V_i_ *and record it as* STD_i_.

**Definition** **3.** **Diff** = {STD_2_-STD_1_, STD_3_-STD_2_, …, STD_n_-STD_n-1_}

Then, we depict the whole STD-TA algorithm as Algorithm 1 shows:
**Algorithm 1.** STD Trend Analysis Method (STD-TA).Input: PA_i−1_, Diff_i−1_, Diff_i_, Diff_i+1_, P_i_, STD_i_, Intrans, Count
If PA_i−1_ ∈ *StaticActivity*:If STD_i_ > 0.1 and max(P_i_) < 0.9:Intrans = 1Count = Count + 1Else:Ɵ_1_ = 1 if Diff_i_* Diff_i−1_ > 0 else 0Ɵ_2_ = 1 if Diff_i+1_* Diff_i_ > 0 else 0Ɵ_3_ = 1 if abs(Diff_i_) > 0.1 else 0Ɵ_4_ = 0.6 if max(P_i_) < 0.6 else max(P_i_)R = 0.5* Ɵ_1_+0.4* Ɵ_2_ + 0.35* Ɵ_3_-(1 − Ɵ_4_)*0.625If R ≥ 1.22:Intrans = 1Count = Count + 1If Intrans == 1:If STD_i_ ≤ 0.1 or max(P_i_) ≥ 0.9 or Count:Intrans = 0Count = 0If Intrans == 1:PA_i_ = TransitionActivityElse:PA_i_ = index(max(P_i_))Output: PA_i_


We refine all activities as Table 4 shows:

After refinement, activities are categorized as two kinds, basic activity and transition activity, and the basic activity mainly consists of static activity (SA) and dynamic activity (DA). Obviously, the SA are long-term stable postures, which means their STD values have few fluctuations.

In our algorithm, we first refer to the previous activity PA_i−1_ of the current window. If it belongs to SA, then we observe STD_i_. If it is a large value and the distribution of probabilistic results is scattered, then we consider the current window as transition activity. If PA_i−1_ ∈ DA, we take 4 factors into consideration: historical trend, future trend, real-time change and probabilistic results. Ɵ_1_ denotes historical trend, and it covers the trend of the STD value of three windows. Ɵ_1_ > 0 means the trend of STD_i-2_, STD_i−1_ and STD_i_ are the same. Ɵ_2_ denotes future trend, which needs to check the next STD value to judge the future trend. Ɵ_2_ > 0 means the trend of STD_i−1_, STD_i_ and STD_i+1_ are the same. Ɵ_3_ is real-time change. When its value increases sharply and overcomes a threshold, it suggests that the current window suffers a huge change with respect to the previous window. Ɵ_4_ denotes probabilistic results. When its value becomes too small, it means that SVM cannot clearly recognize the activity of the current window, which suggests that the current window is likely to be a transition activity. These four factors are a vital basis for judging if the current window is a transition activity. Here we define **Ɵ** = { Ɵ_1_, Ɵ_2_, Ɵ_3_, Ɵ_4_}, and we give the detail determination process of its value in Section 4.

The algorithm finally outputs the recognition result. The transition activities are given the same label, while if it is judged as a basic activity, it will be further classified by SVM to determine its final activity category.

## 4. Experiments

### 4.1. Android Application

**Figure 5:**
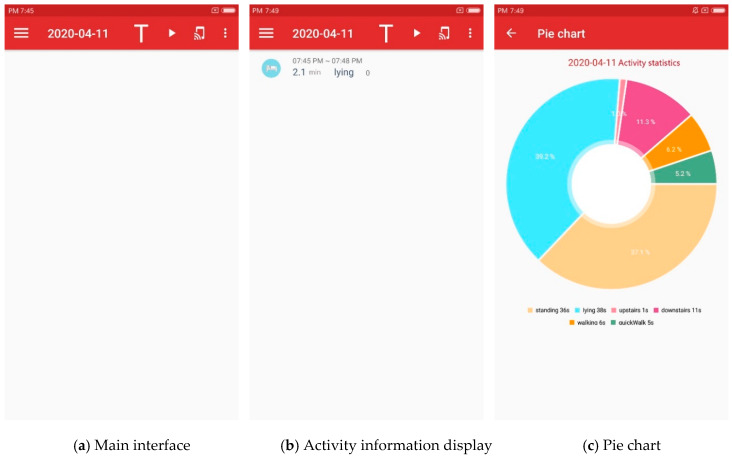

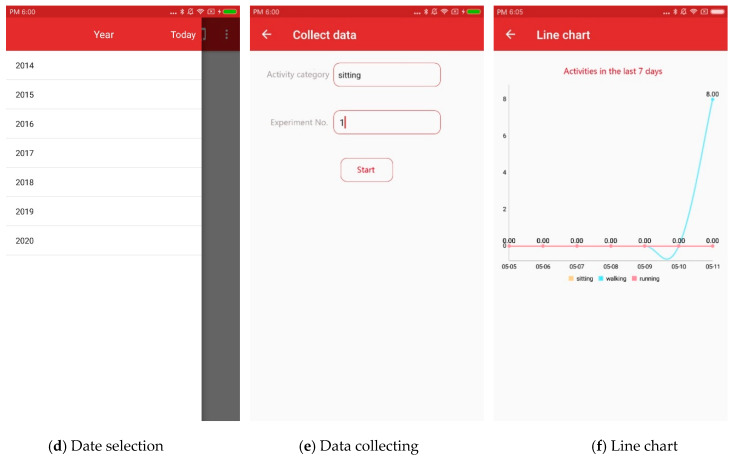
UI of the Android application.

We develop a simple Android application (APP) and deploy it in an Mi phone to conduct our experiments. The APP implements the whole architecture and we deploy it on the phone to carry out data collection and real-time activity recognition. Figure 5 shows the user interface (UI) of APP.

We design this APP to realize the activity recognition and statistics of subjects. We collect activity data using the built-in accelerometer and barometer of a smartphone and recognize the daily activity via the model described in the former sections. Due to the fact that the APP is just in a test stage, the recognition results are stored locally. Meanwhile, we design a long-term monitoring module for users in the APP. The line chart in Figure 5 gives the users’ activities in the previous 7 days, while the pie chart shows the duration and proportion of each daily activity of the users. These functions provide vital context information for high-level applications.

The entire APP is only a test version at present and still needs to be further improved. In the future, we will consider transferring the recognition model and results to the cloud to save the resources of the smart device.

### 4.2. Experiment 1: Classifier Comparison

After data collection and segmentation, we divide the whole dataset into 10 parts, and 10-fold cross validation is carried out on the classifiers. We select three common models, decision tree (DT) [31], K-nearest neighbor (KNN) [32] and support vector machine (SVM) [33], to carry out this experiment and compare the final results. We only consider the characteristics of these classifiers, so here the target activities only include basic activities. Table 5 shows the test results.

According to the results, all classifiers have tremendous high recognition accuracy for static activities (A01–A03), which is nearly 100%. However, this does not mean that it can maintain a high level under real scenarios. For A04, A07 and A08, the accuracy of the three models is all over 95%, while SVM is still slightly better than the other two. Although we combine the data of the accelerometer and barometer, these classifiers are not so effective on A05 and A06, and their accuracy is around 75%. Meanwhile, SVM performs better than the other two on DA. Lastly, we choose SVM as the core classifier in our work.

### 4.3. Experiment 2: Determination of Vector θ

According to Algorithm 1, we recognize the transition activity by analyzing the trend of STD. For SA, its STD value is stable and fluctuates around 0, which is easy to distinguish from DA. However, the boundary between DA and transition activity is confusing, which is the reason why we define the factor vector θ.

**Figure 6:**
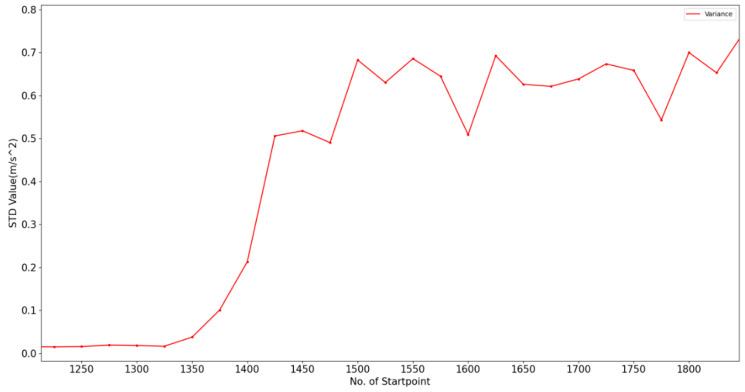
A fragment of “Standing-Walking”.

We take a transition activity as an example. Figure 6 shows the data fragment of “Standing-Walking”.

According to the figure, the vertical axis represents the STD value of the window, and the horizontal axis denotes the starting data point of the window. For example, there is a data point (x = 1150, y = 0.0159) in the figure, which represents the data of a window composed of 50 data points 1150–1200. The STD value of these data is 0.0159.

In the first half of this clip, this subject stays standing, and the STD values of these windows fluctuate around 0 with few changes. When x = 1350, the subject starts to walk. After x = 1450, the subject is completely in walking state. We can clearly detect the change of body posture from the trend of the STD value, especially when x ∈ [1350, 1425]. This is the transition we need to recognize. There are several distinct features of these STD values:

1. Continuous and identical changes. For example, the STD shows a monotonic increasing trend when x ∈ [1350, 1425]. Therefore, for the ith window (i = x/25), we check y_i−1_−y_i−2_, y_i_−y_i−1_ and y_i+1_−y_i_ to judge the current STD trend. If the three values have the same symbol, which means they are all positive or negative, it is likely that the current activity is a transition one. This is what θ_1_ and θ_2_ represent.
2. Huge change. Transition activity is a transition from one stable state to another, so the change between them is usually intense. Thus, for the ith window (i = x/25), we check | y_i_-y_i−1_ |, when its value exceeds the threshold (here we set threshold as 0.1), we judge that the current window may be in transition. This is what θ_3_ represents. It should be noted that the amount of DA data also meets this condition. However, it is still an important indicator of transition activity.
3. Uncertain result. Due to the fact that the transition activity has the same features as multi activities, the recognition result is usually uncertain, although this situation is not absolute. Thus, for the ith window (i = x/25), we check max(P), which is the maximum probability that the current activity belongs to some basic activity. This is what θ_4_ represents.

We extract all the transition activity data fragments and the same amount of basic activity data fragments. For each segment, we calculate the θ as Algorithm 1 depicts. Meanwhile, we set labels for each fragment, 0 for basic activity and 1 for transition activity. Multiple linear regression (MLR) is utilized. The problem is described as follows:

This act denotes current activity, ∀ **W** = {w1,w2,w3,w4},

$$W\theta^{T}=\{\begin{array}{c} 0\;\;\;ThisAct\in Basic\;Activity\;\;\\ 1\;\;ThisAct\in Transition\;Activity \end{array}$$

The function of MLR is finding a proper **W**. With this approach, we get:

**W** = {0.5139511, 0.4159489, 0.35475093, −0.61839164}

After a fine-tuning, we finally determine **W** as Algorithm 1 shows.

### 4.4. Experiment 3: Overall Performance

In this part, we explore the overall performance of our proposed method. For comparison, we set up control groups. Referring to the idea in [34], we regard all transition activities as one activity and assign a unified label to them. We then use SVM and KNN as classifiers for both transition and basic activities. Finally, we do the comparison with our method after experiments.

Here, we first use the whole dataset we acquired before, including basic and transition activities, to train the KNN and SVM. Moreover, we introduce the CUSUM chart (cumulative sum control chart) as another state-of-the-art algorithm in this experiment. CUSUM has excellent performance in change point detection issues [35,36], which are similar with our problem. We use a similar strategy using SVM as the basic classifier and CUSUM as the transition activity recognition method. Then, we invite five new subjects to perform activities as a test set for the four methods. Table 6 gives the results.

According to the results in the table, the three models all perform well on basic activities (A01–A08). Due to the fact that the SAs are stable and easy to distinguish from each other, there are few errors that happen when recognizing them (A01–A03). Accuracy on “Walking” (A04) is relatively low, because as a basic DA, its behavior mode is easily confused with other kinds of DA. In the recognition of “Upstairs” (A05) and “Downstairs” (A06), SVM, STD-TA and CUSUM, which also use SVM as core classifier, have a 6%–10% higher accuracy than KNN. This is similar to our results in Experiment 1. For “Running” (A07) and “QuickWalk” (A08), the results are all at a high level, above 95%. We believe that the intensity of these two activities is tremendously high, which leads to them being quite different from other activities and easy to distinguish.

A09 represents all transition activities. Both SVM and KNN maintain an accuracy of 66%. Obviously, a number of pieces of data which belong to the transition activity category are mistakenly identified as basic activities, and vice versa. We infer that transition activity has high similarity with DA. The CUSUM achieves a recognition accuracy of 73.24%. It is effective for detecting the change point of the activity sequence. However, this method is effective for a single kind of transition activity, which has limitation on recognizing multiple kinds of transitions in real scenarios. In contrast, our method improves the accuracy of detection of transition activity by nearly 20%, reaching 80%. It is supposed to be the trend features extracted by the STD-TA that produces the promotion.

## 5. Conclusions and Future Work

HAR is faced with some problems, and the transition activity mentioned in this paper is one of them. To solve this issue, we designed an algorithm based on STD trend analysis. For basic activity, we mainly utilized SVM for recognition. For transition activity, we analyzed the STD value of data to judge the trend of the overall data flow to recognize the activity. Through result comparison, the accuracy of our algorithm is 20% higher, reaching 82.85%, than only using a machine learning model in identifying the transition activity.

Our work has achieved promising results in distinguishing transition activity from basic activity. However, our method does not have an absolute advantage over the other two classifiers in the control group on distinguishing between transition activity and walking. In addition, the generalization of some specific parameters we calculate in this paper, such as **W** and θ, is limited. If there were some users whose height or age is beyond the range of the training set, the accuracy would be affected. Therefore, we should design an incremental update mechanism for these parameters in our future work. Moreover, we do not further discuss the specific categories of transition activities. This is because that we can judge the specific type of transition activity through activities before and after it. Adding the specific recognition method could increase the complexity of the whole architecture. In future work, we will focus on the generalization of our model and discuss more complex daily activities (e.g., cooking, reading, etc.).

## Figures and Tables

**Table 1:** Information of our dataset.

**Basic Activity**		Sitting, Standing, Lying, Walking, Upstairs, Down Stairs, Running, QuickWalk
**Sensors (Sample Frequency)**		1 Barometer (5 Hz), 1 3D-Accelerometer (50 Hz)
**Subjects**	**No. of Subjects**	10
	**Age Range**	25–40
	**Male/Female**	7/3
	**Height Range**	155 cm~180 cm
**Time of Single Collection**		3 min
**Size of Time Window**		1 s (50 data points)
**Overlap**		0.5 s (25 data points)

**Table 2:** Duration of each activity.

Activity	Time (Data Point)
downstairs	50–60
quickwalk	50–60
upstairs	50–60
walking	50–60
running	30–50
downstairs↔standing	50
lying↔sitting	160
quickwalk↔standing	30
sitting↔standing	150
upstairs↔standing	40
standing↔walking	45
walking↔quickwalk	10–20
walking↔running	10–20

**Table 3:** Duration of each activity.

No.	Feature	Formula
1	Mean	$\bar{a}$
2	Variance	$\overset{n}{\underset{i=1}{\sum }}{\left(a_{i}-\mu \right)}^{2}$
3	STD	$\sqrt{\frac{\sum_{i=1}^{n}{\left(a_{i}-\mu \right)}^{2}}{n}}$
4	Maximum	$max\left(a_{i}\right)$
5	Minimum	$min\left(a_{i}\right)$
6	Range	$max\left(a_{i}\right)-min\left(a_{i}\right)$
7	ZCR	$\overset{n}{\underset{i=1}{\sum }}sig(a_{i}>0)$
8	Median	$median\left(a_{i}\right)$
9	MAD	$median(|a_{i}-median\left(a_{i}\right)|)$
10	Information Entropy	$-\overset{m}{\underset{i=1}{\sum }}\left(p_{i}*\log\left(p_{i}\right)\right)$
11	Kurtosis	$E\left[{\left(a_{i}-\mu \right)}^{4}/\sigma^{4}\right]$
12	Skewness	$E\left[{\left(a_{i}-\mu \right)}^{3}/\sigma^{3}\right]$
13	Coefficient	cov(X,Y)

ZCR: zero crossing rate MAD: absolute median difference.

**Table 4:** Detailed category of activities.

**Activity**	Basic Activity	Static Activity	Sitting Standing Lying
		Dynamic Activity	Walking Upstairs Downstairs Running QuickWalk
	Transition Activity	Lying-Standing Sitting-Standing Standing-Upstairs Standing-Downstairs Standing-Walking Walking-Running Walking-QuickWalk	

**Table 5:** Recognition results comparison between three classifiers.

DT	A01	A02	A03	A04	A05	A06	A07	A08
**A01**	703	0	0	0	0	0	0	0
**A02**	0	745	0	0	0	0	0	0
**A03**	0	0	711	0	0	0	0	0
**A04**	0	0	0	664	4	11	0	14
**A05**	0	0	0	18	478	64	0	4
**A06**	0	6	0	33	63	339	0	3
**A07**	0	0	0	1	0	0	688	0
**A08**	0	0	0	4	1	1	0	692
**KNN**	**A01**	**A02**	**A03**	**A04**	**A05**	**A06**	**A07**	**A08**
**A01**	703	0	0	0	0	0	0	0
**A02**	0	745	0	0	0	0	0	0
**A03**	0	0	711	0	0	0	0	0
**A04**	0	0	0	679	4	4	0	6
**A05**	0	0	0	48	484	22	0	10
**A06**	0	0	0	51	39	351	0	3
**A07**	0	0	0	0	0	0	689	0
**A08**	0	0	0	1	4	0	0	693
**SVM**	**A01**	**A02**	**A03**	**A04**	**A05**	**A06**	**A07**	**A08**
**A01**	703	0	0	0	0	0	0	0
**A02**	0	745	0	0	0	0	0	0
**A03**	0	0	711	0	0	0	0	0
**A04**	0	0	0	684	1	3	0	5
**A05**	0	0	0	12	516	30	0	6
**A06**	0	6	0	15	39	381	0	3
**A07**	0	0	0	0	0	0	689	0
**A08**	0	0	0	1	2	0	0	695

A01–A08 denote basic activities “sitting, standing, lying, walking, upstairs, downstairs, running, quickwalk”.

**Table 6:** Overall performance of four methods.

SVM	A01	A02	A03	A04	A05	A06	A07	A08	A09	SUM	Recall
**A01**	6552	34	0	44	0	0	0	0	0	6630	0.988235
**A02**	0	6417	25	33	0	0	0	0	0	6475	0.991042
**A03**	5	5	6510	0	0	0	0	0	0	6520	0.998466
**A04**	0	2	114	5621	110	123	84	125	246	6425	0.874864
**A05**	0	0	0	243	5966	117	43	19	27	6415	0.930008
**A06**	0	0	0	108	123	5852	120	86	196	6485	0.90239
**A07**	0	0	0	32	0	13	6354	56	0	6455	0.984353
**A08**	0	0	0	54	51	25	71	6146	66	6413	0.958366
**A09**	0	0	0	352	186	174	76	213	1944	2945	0.660102
**KNN**	**A01**	**A02**	**A03**	**A04**	**A05**	**A06**	**A07**	**A08**	**A09**	**SUM**	**Recall**
**A01**	6617	0	8	5	0	0	0	0	0	6630	0.998039
**A02**	0	6475	0	0	0	0	0	0	0	6475	1
**A03**	4	12	6504	0	0	0	0	0	0	6520	0.997546
**A04**	0	67	19	5552	76	114	24	169	404	6425	0.864125
**A05**	0	0	22	188	5644	139	213	114	95	6415	0.879813
**A06**	0	0	13	355	345	5124	213	267	168	6485	0.790131
**A07**	0	0	0	51	67	0	6289	41	7	6455	0.974284
**A08**	0	0	0	19	0	0	12	6312	70	6413	0.984251
**A09**	0	0	0	220	206	162	167	245	1945	2945	0.660441
**STD-TA**	**A01**	**A02**	**A03**	**A04**	**A05**	**A06**	**A07**	**A08**	**A09**	**SUM**	**Recall**
**A01**	6618	4	8	0	0	0	0	0	0	6630	0.99819
**A02**	0	6475	0	0	0	0	0	0	0	6475	1
**A03**	22	41	6457	0	0	0	0	0	0	6520	0.990337
**A04**	0	0	22	6014	67	0	122	67	133	6425	0.936031
**A05**	0	0	0	154	5848	46	77	41	249	6415	0.911613
**A06**	0	0	0	188	205	5622	169	105	196	6485	0.866924
**A07**	0	0	0	10	4	0	6375	22	44	6455	0.987607
**A08**	0	0	0	70	14	0	42	6254	33	6413	0.975207
**A09**	0	0	7	122	105	91	78	25	2517	2945	0.854669
**CUSUM**	**A01**	**A02**	**A03**	**A04**	**A05**	**A06**	**A07**	**A08**	**A09**	**SUM**	**Recall**
**A01**	6603	17	10	0	0	0	0	0	0	6630	0.995928
**A02**	0	6475	0	0	0	0	0	0	0	6475	1
**A03**	6	3	6511	0	0	0	0	0	0	6520	0.99862
**A04**	0	21	1	5741	72	72	88	76	354	6425	0.893541
**A05**	0	0	12	121	5798	139	109	114	122	6415	0.903819
**A06**	0	0	13	241	345	5336	154	267	129	6485	0.822822
**A07**	0	0	0	25	44	57	6300	24	5	6455	0.975988
**A08**	0	0	2	31	20	40	51	6147	122	6413	0.958522
**A09**	0	0	0	120	206	162	167	133	2157	2945	0.732428
